# Structural Insights into the Abscisic Acid Stereospecificity by the ABA Receptors PYR/PYL/RCAR

**DOI:** 10.1371/journal.pone.0067477

**Published:** 2013-07-02

**Authors:** Xingliang Zhang, Lun Jiang, Guoqiang Wang, Lin Yu, Qi Zhang, Qi Xin, Wei Wu, Zhizhong Gong, Zhongzhou Chen

**Affiliations:** 1 State Key Laboratory of Agrobiotechnology, China Agricultural University, Beijing, China; 2 Clinical Medicine Research Center, Affiliated Hospital of Guangdong Medical College, Guangdong, China; 3 State Key Laboratory of Plant Physiology and Biochemistry, China Agricultural University, Beijing, China; Van Andel Research Institute, United States of America

## Abstract

The phytohormone abscisic acid ((+)-ABA) plays a key role in many processes. The biological and biochemical activities of unnatural **(−)**-ABA have been extensively investigated since 1960s. However, the recognition mechanism by which only a few members among PYR/PYL/RCAR (PYLs) family can bind **(−)**-ABA remains largely unknown. Here we systematically characterized the affinity of PYLs binding to the **(−)**-ABA and reported the crystal structures of apo-PYL5, PYL3-**(−)**-ABA and PYL9-(+)-ABA. PYL5 showed the strongest binding affinity with **(−)**-ABA among all the PYLs. PYL9 is a stringently exclusive (+)-ABA receptor with interchangeable disulfide bonds shared by a subclass of PYLs. PYL3 is a dual receptor to both ABA enantiomers. The binding orientation and pocket of **(−)**-ABA in PYLs are obviously different from those of (+)-ABA. Steric hindrance and hydrophobic interaction are the two key factors in determining the stereospecificity of PYLs binding to **(−)**-ABA, which is further confirmed by gain-of-function and loss-of-function mutagenesis. Our results provide novel insights of the bioactivity of ABA enantiomers onto PYLs, and shed light on designing the selective ABA receptors agonists.

## Introduction

Abscisic acid ((+)-ABA) is indispensable for a great many processes such as root growth, stomatal aperture, seed maturation, dormancy, and response to abiotic stresses including drought, cold and salinity [1], [2], [3]. A 13-member family of ABA receptors PYR/PYL/RCAR (PYLs) in *Arabidopsis thaliana* was genetically and biochemically identified [4], [5]. Based on the previously solved structures PYL1, PYL2, and PYR1, an ABA-dependent signaling mechanism was proposed by several groups [6], [7], [8], [9], [10]. Upon binding (+)-ABA, dimeric PYLs underwent a pronounced conformational rearrangement, and then bound and inhibited the group A protein phosphatases type 2Cs (PP2C) [6], [7], including ABI1, ABI2, HAB1 and PP2CA. PYLs/PP2C heterodimer blocked substrates binding to PP2Cs, and thus released downstream transcription factors SnRK2 kinases formerly inhibited by PP2C. In addition, according to different intrinsic affinities of PYLs to ABA, two distinct classes of receptors, dimer and monomer, were identified [11]. In our previous study, it was demonstrated that PYL3 formed a unique trans-dimer intermediate upon binding (+)-ABA or pyrabactin [12].

**Figure 1:**
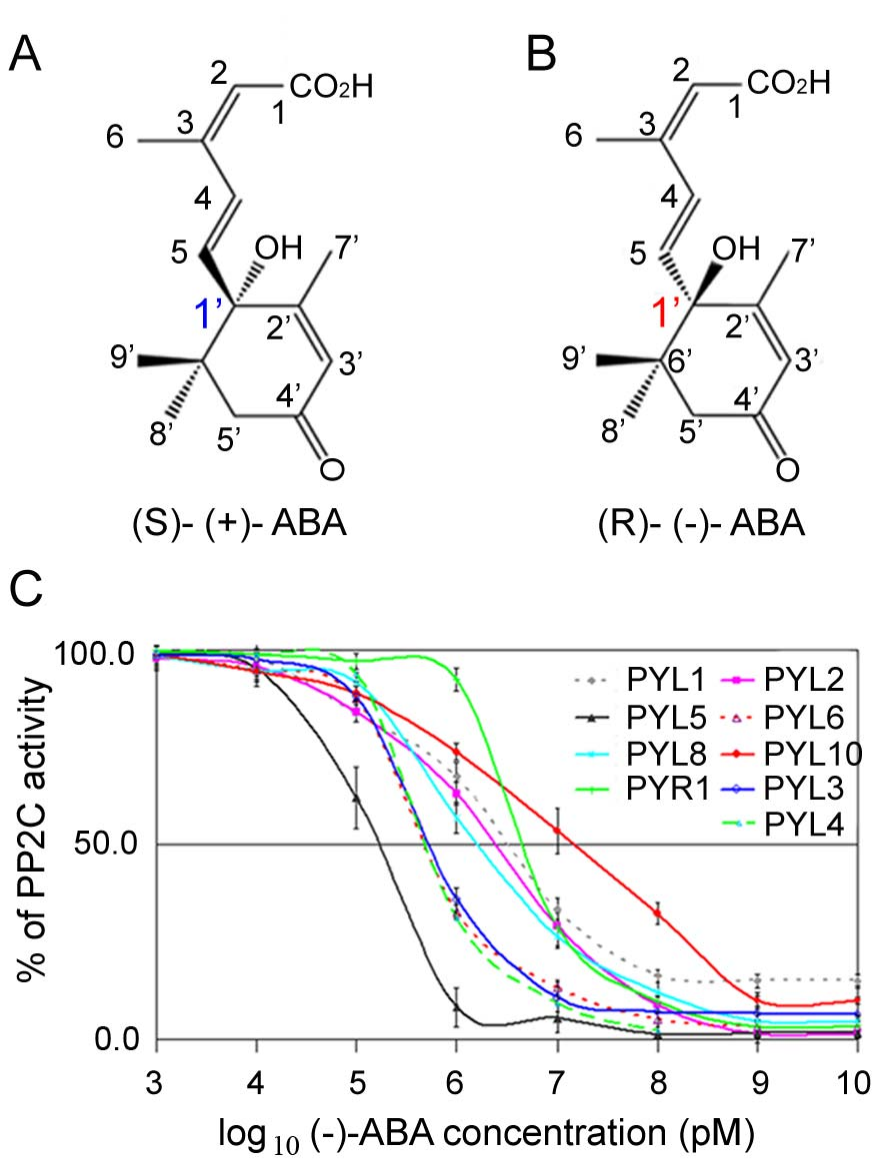
Structure and biochemical characterizations of (−)-ABA. Structures of (+)-ABA (**A**) and **(−)**-ABA (**B**). (**C**) **(−)**-ABA mediated inhibition of HAB1 phosphatase activity by PYLs. The concentration for each PYLs protein was 5.0 µM and for HAB1 was 3.0 µM. All experiments were repeated three times (n = 3) and error bars represented s.d. The condition measuring the phosphatase activity of HAB1 was same below unless noted.

The naturally occurring form is S-(+)-ABA (hereafter referred to as (+)-ABA), with one asymmetric carbon atom at C-1′ position (Fig. 1A). Nevertheless, the biological and biochemical activities of synthetic enantiomer **(−)**-ABA (Fig. 1B) have been extensively investigated since the discovery of ABA [3], [13]. In previous studies such as stomatal closure, **(−)**-ABA is weakly active. But, in some other assays including seed germination [14] and plant tissues growth [13], the applied **(−)**-ABA has been found to have comparable activity to (+)-ABA. Furthermore, microarray and genetic studies have shown that a functional ABA signaling pathway is required for **(−)**-ABA activity in *Arabidopsis* [14], [15]. In addition, **(−)**-ABA can induce the biosynthesis of (+)-ABA in plant tissues [16]. Because **(−)**-ABA is lower membrane permeable and slower metabolized than natural (+)-ABA *in vivo* [17], it may even have stronger activity than (+)-ABA at the same concentration [16].

To explain the bioactivity of **(−)**-ABA, two mechanisms were hypothesized, one was the same occupied site between **(−)**-ABA and (+)-ABA through flipping the cyclohexene plane [13], the other was the dual selectivity of ABA receptors [14]. The preliminary crystallographic studies of PYR1 with enantiomorphic (±)-ABA mixture provided an explanation for “flip” hypothesis which proposed that the **(−)**-ABA ring was flipped ∼180° from the (+)-ABA ring [10]. However, according to the yeast two-hybrid data [5], the interaction between PYR1 and HAB1 was very weak in the presence of **(−)**-ABA, which was opposite with (+)-ABA. Therefore, both ABA enantiomers in the one crystal [10] made **(−)**-ABA’s information inaccurate due to its lower and uncertain occupancy, which compromised its value for detailed analysis. Based on the binding between (+)-ABA and PYL2, the arrangement of the hydrophobic residues surrounding the cyclohexene moiety was supposed to underlie the stereoselectivity of ABA isomers [6], [18]. However, the orientation of **(−)**-ABA in the pocket of PYLs and the base for the stereospecificity of PYLs bind to both ABA enantiomers are still unclear. Considering PYL5/RCAR8 and PYL8/RCAR3, but not PYL9/RCAR1, respond to both enantiomers [5], [19], PYLs family may contain some candidates responsible for the dual selectivity. To date, none of the solved structures from protein data bank is found to contain **(−)**-ABA. It has not yet been clear why the stereoselectivity was different for PYLs and there was no direct evidence to show a dual selectivity of PYLs binding both ABA enantiomers.

To elucidate the above questions, we solved the structures of apo-PYL5, PYL9-(+)-ABA and PYL3-**(−)**-ABA. Biochemical and structural observations indicated that several PYLs, at least PYL3, could bind **(−)**-ABA to inhibit PP2C activity. Moreover, a disulphide bond observed in PYL9-(+)-ABA structure might be shared with a subclass composed of PYL4 and PYL6-10. Structural analysis along with biochemical data *in vitro* demonstrated that steric hindrance and hydrophobic interaction in the pocket of PYLs were crucial for their stereospecificity to ABA enantiomers.

## Materials and Methods

### Protein Preparation

PYR1 and PYL1 to PYL12 were subcloned from the *Arabidopsis thaliana* cDNA library (Fig. S1) using standard PCR-based protocol. All mutants were generated with two-step PCR. Except PYL3, the cDNA sequences corresponding to the WT and all mutants of PYLs were inserted into the pET-28a or pGEX-4T-2, in which the thrombin recognition site was replaced by TEV recognition site. The inserted sequence was verified by DNA sequencing, and transformed into *Escherichia coli* strain BL21 (DE3) for protein expressions. Transformed cells were then cultured at 37°C in LB medium containing 50 µg/ml kanamycin or ampicllin. When the culture density reached an OD_600_ of 0.8–1.0, induction with 0.1 mM IPTG was performed, and cell growth continued for an additional 24 hours at 18°C. Cells were harvested by centrifugation at 3, 000 g for 15 min, and then resuspended in Lysis Buffer (20 mM Tris-HCl pH 8.0, 150 mM NaCl, 0.1 mM DTT) and lysed by sonication. The lysate was centrifuged at 47,000 g for 20 min and the supernatant was filtrated by 0.45 µm filter membrane to remove cell debris and other impurities, and then applied to Profinity™ IMAC Ni-Charged Resin column (Bio-Rad), then further purified by size exclusion chromatography (Superdex 200 HR10/300 GL, GE Healthcare).

HAB1 (residues 169–511) was inserted into the pGEX-4T-2 vector in which the thrombin recognition site was also replaced by TEV recognition site and the sequence of the insert was verified by DNA sequencing. The culture, expression, harvest, sonication and centrifugation were the same to those of PYLs. The supernatant after filtration by 0.45 µm filter membrane was applied to Glutathione Sepharose 4 FF Resin column (GE Healthcare). Then this column was washed with twenty-fold bed volume Lysis Buffer. To excise GST tag, a small amount of 6×His tagged TEV protease was added into this column and incubated on ice overnight. The digestion production went through Profinity™ IMAC Ni-Charged Resin column (Bio-Rad) to remove TEV protease. The flow through underwent a further purification step of size exclusion chromatography (Superdex 200 HR10/300 GL, GE Healthcare, GE Healthcare).

### Crystallization and Data Collection

To get the PYL9-(+)-ABA complex crystals, (+)-ABA was mixed with 6×His-tagged PYL9 at 5∶1 ratio and then incubated for 2–3 hours on ice. The mixture was applied to size exclusion chromatography and the fractions corresponding to the peak of homogeneous complex were merged and concentrated to about 20 mg/ml. The crystallization screen conditions were from commercial kits (Hampton Research and Emerald Biosystems) and some home-made products. Initial trails were performed at 20°C by sitting-drop vapor diffusion method. Crystallization-solution droplet was comprised of 1.0 µl each reservoir solution and 1.0 µl freshly purified target protein or complex, which was equilibrated against 100 µl each reservoir solution. The complex crystals appeared in a well solution contained 30% PEG 4000, 0.1 M Tris-HCl pH 8.3, 0.2 M Li_2_SO4. The crystal was transferred into well solution containing 30% glycerol as cryo-protectant solution and flash-cooled in liquid nitrogen before collecting data.

Purified 6×His-tagged PYL5 was concentrated to about 20 mg/ml for screening crystal. The manipulation of apo-PYL5 crystallization experiments was the same as that of PYL9-(+)-ABA. Apo-PYL5 native crystal appeared after 2–3 days in the reservoir solution contained 13% PEG 3000, 0.2 M Ca(Ac)_2_, 0.1 M Tris-HCl pH 7.4, 5% glycerol.

To obtain PYL3-**(−)**-ABA complex crystal, the fragments of PYL3 were screened to be appropriate for complex crystallization. **(−)**-ABA was mixed with PYL3 at 10∶1 ratio and stayed on ice overnight. PYL3-**(−)**-ABA complex were concentrated to about 20 mg/ml. The manipulation of crystallization experiments was the same as above. Finally, crystals of PYL3 (residues 21–209) complexed with **(−)**-ABA were grown in the well buffer containing 20% PEG 8000, 0.1 M Tris-HCl pH 8.5, 0.2 M MgCl_2_.

All the crystal data were collected at KEK beamline NE3A, SSRF beamline BL17U and BSRF beamline 1W2B. All the data were integrated and scaled with the HKL2000 suite of programs [20]. Data collection statistics were summarized in Table 1.

**Table 1:** Data collection and refinement statistics of PYLs and complexes.^*^

	Apo-PYL5	PYL9-(+)-ABA	PYL3-(−)-ABA
**Data collection**			
Space group	*P*6_4_	*P*3_2_	*P*6_5_
Cell dimensions			
* a*, *b*, *c*, Å	95.9, 95.9,144.0	112.0, 112.0, 40.4	142.7, 142.7, 99.8
α, β, γ, °	90, 90, 120	90, 90, 120	90, 90, 120
Resolution, Å	50.0–2.60 (2.64–2.60)†	50.0–2.68 (2.73–2.68)†	50.0–2.65 (2.70–2.65)†
*R*merge, %	7.8 (39.5)	8.4 (39.3)	8.1 (42.5)
*I*/σ*I*	33.9 (3.1)	15.9 (2.3)	24.0 (2.3)
Completeness, %	95.7 (81.0)	99.5 (95.2)	95.1 (70.8)
Redundancy	12.3 (6.9)	6.3 (3.5)	4.1 (3.5)
**Refinement**			
Resolution, Å	40.0–2.60 (2.67–2.60)	50.0–2.68 (2.75–2.68)	50.0–2.65 (2.72–2.65)
No. of reflections	20210 (1082)	15081 (796)	29323 (1544)
*R*_work_/*R*_free_, %	25.3/27.4 (32.6/48.0)	17.7/20.4 (32.2/32.0)	24.3/26.7 (34.7/48.5)
No. of atoms			
Protein	3200	2555	5225
Ligand/ion	72	38	76
Water	565	231	95
*B*-factors			
Protein	49.95	33.99	67.07
Ligand/ion	36.67	11.53	62.54
Water	31.03	38.58	66.53
rms deviations			
Bond lengths, Å	0.008	0.009	0.008
Bond angles, °	1.444	1.255	1.287
Ramachandran Plot, %^2^	97.8/2.2/0	100/0/0	99.7/0.3/0

† Statistics for highest resolution shell. ^*^Three crystal experiments for each structure.

2 Residues in favored, generously allowed and disallowed regions of the Ramachandran plot.

### Structure Determination

Using our apo-PYL3 structure (PDB code 3KLX) as the search model, molecular replacement solutions of the PYL9-(+)-ABA and apo-PYL5 and PYL3-**(−)**-ABA were found using PHASER [21] and MOLREP [22]. The R factor was improved by TLSMD analysis [23]. The data of apo-PYL5 were determined to be merohedral-twinned, as judged by cumulative intensity distribution calculated with the program Phenix [24]. We used the program Refmac5 [25] to refine the structure. The model was built manually in the program COOT and CCP4 package. Structure refinement statistics were shown in Table 1.

### Phosphatase Activity Assay

The phosphatase activity was measured by the serine-threonine phosphatase assay system (Promega V2460). Each reaction was performed in a 50 µl reaction buffer (20 mM Hepes pH 7.5 and 150 mM NaCl) containing 0.5 µM HAB1, 1 µM WT or mutant PYL3, PYL5 or PYL9 protein and (+)-ABA or **(−)**-ABA of 5 µM concentration if required. 30 min later at room temperature, 5 µl phosphorylated peptide substrate supplied with the Promega kit was added into the reaction system at 30°C for 25 min. And then the reaction was terminated by addition of 50 µl molybdate dye/additive mixture, and the absorbance at 620 nm was measured 30 min later. The OD_620_ value of the reaction without HAB1 was set as baseline while the phosphatase activity of the reaction without PYLs was set as 100% for HAB1. Each reaction was repeated at least three times and the error bars indicated standard deviations.

### Isothermal Titration Calorimetry (ITC) Assays

PYL3 protein was subjected to size exclusion chromatography with Buffer A (20 mM Tris-HCl pH 8.0 and 150 mM NaCl). 100 mM ABA was dissolved in buffer A and adjusted to pH 8.0 with NaOH. ABA sample for experiment was obtained by diluting 100 mM stock solution with buffer A. 3 mM (±)-ABA (Sigma) or 0.3 mM **(−)**-ABA (Sigma) or only Buffer A in the syringe was titrated against 0.22 mM PYL3 in the cell, respectively. The data from only Buffer A injection to PYL3 was taken as control. The temperature of reaction system was constantly set to 20°C. The experiments were performed with MicroCal iTC200 or Nano ITC. Thermodynamic parameters were determined by software origin.

Details of analytical ultracentrifugation, cross-linking gel assay, size exclusion chromatography and GST-mediated pulldown assay were described in File S1.

## Results

### Systematic Characterizations of PYLs Inhibiting HAB1 in the Presence of **(−)**-ABA

To address the stereospecificity of **(−)**-ABA dependent inhibition of PP2Cs by PYLs, we characterized PYLs proteins systematically. Except that PYL7, PYL11 and PYL12 formed inclusion body in *E. coli*, all functional PYLs from *Arabidopsis thaliana* were purified to homogeneity. All the tested PYLs inhibited the phosphatase activity of PP2Cs mediated by (+)-ABA. Then we examined their ability to inhibit the activity of HAB1 in the presence of **(−)**-ABA. The **(−)**-ABA concentration required to achieve 50% inhibition (IC_50_) of HAB1 activity was 4.5, 3.1, 2.5, 0.51, 0.48, 0.17, 0.47, 1.6 and 15 µM in the presence of PYR1, PYL1, PYL2, PYL3, PYL4, PYL5, PYL6, PYL8 or PYL10, respectively (Fig. 1C). By contrast, PYL9 did not inhibit PP2C activity at 10 µM or even higher concentration of **(−)**-ABA. Our previous studies divided PYLs into three subclasses according to conformations of ligand-bound PYLs [12]. Accordingly, in the presence of **(−)**-ABA, the inhibition of HAB1 by monomeric PYLs was stronger than that by cis-dimeric PYLs, except monomeric PYL10 with a higher IC_50_ and broad range of inhibitory concentration. Interestingly, trans-dimeric PYL3 showed a powerful inhibition of PP2Cs like monomeric PYLs. The results implied PYL5 might bind **(−)**-ABA very strong, followed by PYL3, PYL4 and PYL6 with the same level (Fig. 1C). In contrast, PYL9 could not bind **(−)**-ABA. PYL5 and PYL9 represent two extremes of recognizing **(−)**-ABA. Thus we need further structural and biochemical investigations of them. After extensive trials, we successfully determined structures of PYL9-(+)-ABA, apo-PYL5, and PYL3-**(−)**-ABA at high resolution.

### PYL9 is a (+)-ABA Receptor with Interchangeable Disulfide Bonds

**Figure 2:**
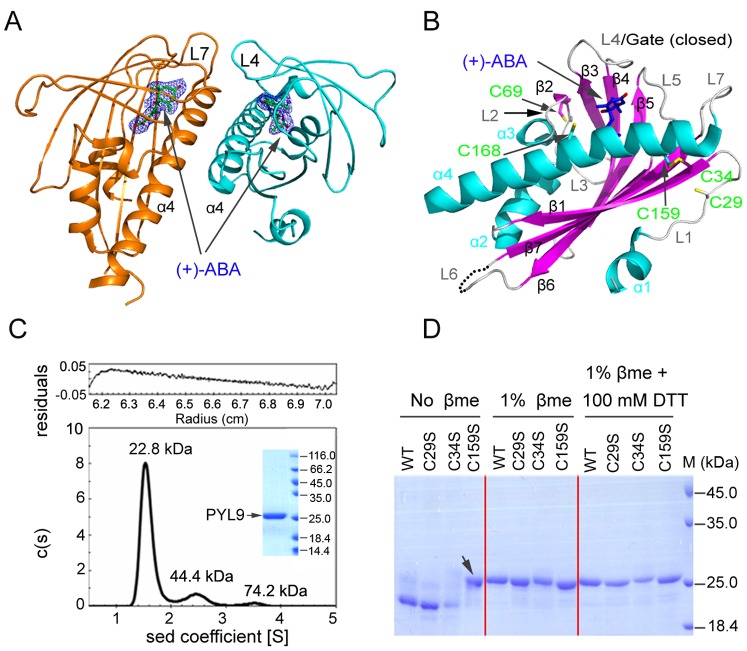
Structural characterizations and bioactivity of PYL9-(+)-ABA. (**A**) Two protomers of PYL9-(+)-ABA in each asymmetric unit. 2*F_o_*−*F_c_* electron density map of (+)-ABA at 1.0σ. (**B**) One protomer of PYL9-(+)-ABA with five cysteine residues (green) and a disulphide formed between C34 and C159. (**C**) The monomeric state of PYL9 in solution was confirmed by the sedimentation velocity. The sample purity for sedimentation velocity experiments was detected by SDS-PAGE and then Coomassie Brilliant Blue staining in the subplot. (**D**) The C159 was the key residue in disulphide bond, while the C29 competed with the C34 to form the disulphide bond. PYL9 and its mutants were under different oxidation-reduction conditions for SDS-PAGE. 1% βme or 1% βme +100 mM DTT was used as reducing agents to disrupt the disulphide bond. Only C159S mutant was not affected by the reducing agents (black arrow).

As an extreme of excluding **(−)**-ABA binding, PYL9 has fueled our interest in its structures. In addition, protein structure database search revealed that no structure from subfamily I [4] has been reported. To know why PYL9 can not bind **(−)**-ABA, we tried to solve the structures. Crystallization trials of PYL9 without hormone were unsuccessful. Luckily, we succeeded in generation of diffraction-quality crystals (Fig. S2A). The complex of PYL9 with (+)-ABA was solved at 2.68 Å resolution (Table 1). Each asymmetric unit contained a symmetric cis-dimer (Fig. 2A). One protomer had four α-helices and seven β-sheets (Fig. 2B), sharing the similar arrangements of other known PYLs. Loops L4 and L5 played the role of the gate and the latch respectively, which locked the (+)-ABA in a conserved pocket [6]. The (+)-ABA was encaged in the cavity by abundant hydrophobic interactions, several hydrogen bonds and two salt bridges (Fig. S2B). Besides, PYL9 can inhibit HAB1 in the presence of (+)-ABA (Fig. S3A). Therefore these results revealed that PYL9 was a (+)-ABA receptor.

The above structural analysis indicated that PYL9 existed as a cis-dimer in the crystal and had a large interface area (Fig. 2A), like PYL2. To characterize whether the cis-dimer was physiological or a crystal packing artifact, PYL9 was subjected to size exclusion chromatography (Fig. S2C) and cross-linking gel assay (Fig. S2D). The results showed that PYL9 was mainly in monomeric form, unlike dimeric PYL1–3 and PYR1. Compared with other PYLs, a distinct phenomenon was the random aggregation of PYL9, which provoked our interest in further investigation since the homogeneity of protein was vital for crystallization. The monomeric fraction of PYL9 was subjected to size exclusion chromatography once again (Fig. S2E) and then checked by sedimentation velocity experiment (Fig. 2C). These results showed that no obvious aggregation for monomeric form of PYL9, indicating that aggregation was not equilibrated.

Surprisingly, a disulphide bond between C34 and C159 was observed in PYL9-(+)-ABA structure (Fig. 2B). Besides, the C29 on flexible loop L1 located near the disulphide bond. To check whether the C29 would disturb the disulphide bond formation and mutation would give any impacts on PYL9 activity, the homogeneity of wild type and C29S, C34S and C159S mutants were detected by non-reduced and reduced SDS-PAGE (Fig. 2D). The homogeneity of C159S was not affected by reducing condition, indicated that C159 was the key residue in disulphide bond. Overall, the homogeneity of the C29S and C34S mutants resembled wild type, showing that the C29 competed with the C34 to form a disulphide bond with the C159 in solution. It implies that disulfide bonds can form either between residues 159 and 29, or 159 and 34.

The position of (+)-ABA in the PYL9-(+)-ABA structure is different from that in the PYL3-(+)-ABA structure [12]. Therefore, the binding of (+)-ABA are diverse and we can not deduce the exact position of **(−)**-ABA from the PYLs-(+)-ABA structures and PYLs sequences (Fig. S1). We next focused on the study of the complex of PYLs and **(−)**-ABA.

### PYL3 has a Dual Selectivity

**Figure 3:**
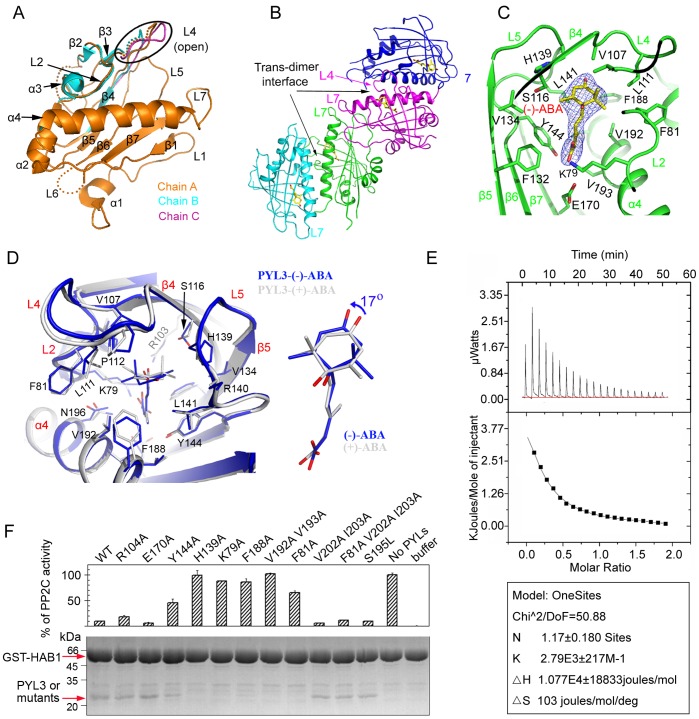
Characterizations of PYL5 and PYL3-(−)-ABA. (A). Chain A together with L2 of chain B and L4 of chain C displayed a whole protomer of apo-PYL5 (also seen Fig. S4C). (**B**) Four PYL3-**(−)**-ABA molecules in an asymmetric unit, organized as two trans-dimers. (**C**) Close-up view of **(−)**-ABA (yellow) in the binding pocket. 2*F_o_* − *F_c_* electron density map of **(−)**-ABA at 1.0σ. (**D**) Superposition of PYL3-**(−)**-ABA and PYL3-(+)-ABA (PDB: 4DSC). There were partially rotation and shift between the rings in both ABA. **(−)**-ABA was constrained in a hydrophobic cavity with little flexibility in PYL3. (**E**) The binding affinity of **(−)**-ABA to PYL3, assessed by ITC assays, was less than that of (+)-ABA or (±)-ABA, (also seen Fig. S5D,E). (**F**) PP2C activity (upper panel) and GST-mediated pulldown of PYL3 mutants protein in the presence of **(−)**-ABA (lower panel). GST-HAB1 and PYL3, highlighted by red arrows, were visualized by Coomassie Brilliant Blue staining.

PYL5 bound **(−)**-ABA most strongly (Fig. 1C), so we first tried to acquire the complex crystal of PYL5 with **(−)**-ABA. However, the X-ray diffraction data showed that it was highly twinned and could not be merged together. In addition, X-ray diffraction images from apo-PYL5 crystal also showed a highly twin (Fig. S4A). Further attempts to improve the crystals did not succeed. Nevertheless, the apo-PYL5 structure was solved at a resolution of 2.68 Å (Table 1) (Fig. 3A, S4B).

Other PYLs with different fragments were screened for PYLs-**(−)**-ABA complex crystals. Finally, only crystal from residues 21–209 of PYL3 complexed with **(−)**-ABA generated diffraction-quality data. PYL3-**(−)**-ABA complex structure was solved at 2.65 Å resolution (Table 1). There were two PYL3-**(−)**-ABA dimers in an asymmetric unit (Fig. 3B). Interestingly, the dimer in this complex was a trans-dimer (Fig. 3B), which was in agreement with PYL3-(+)-ABA and PYL3-pyrabactin structures [12]. Therefore, trans-dimer conformation is common for ligand-bound PYL3. The **(−)**-ABA position was found by the clear F*_o_*-F*_c_* difference electron density map and further confirmed by the low thermal factors (Fig. S5A) and 2F*_o_*-F*_c_* electron density map (Fig. 3C). The protomer structure in the PYL3-**(−)**-ABA complex was similar to the previously reported PYL3-(+)-ABA structure with a root-mean-square deviation of 0.7 Å between their Cα atoms. Besides, the closed loop L4 locked the ligand **(−)**-ABA into the binding pocket.

However, there were several obvious differences between these two complexes. First, the cyclohexene ring in **(−)**-ABA rotated anticlockwise by 17° (right panel of Fig. 3D), compared with that in (+)-ABA. Second, H139 and L141 in the loop L5 of the PYL3-**(−)**-ABA complex (Fig. 3D) moved 0.8 Å away to leave room for adopting the rotating hydroxyl group of **(−)**-ABA. The L111 in the loop L4 and F188 in the α4 of the PYL3-**(−)**-ABA complex moved 0.7 Å away to extend the binding pocket to adopt the 8′ and 9′ dimethyl groups of **(−)**-ABA. Third, 7′ methyl group of **(−)**-ABA moved 0.3 Å closer to the β4, which was adjacent to several hydrophobic amino acids, such as V134 in β5 (Fig. 3D). The carboxyl group of **(−)**-ABA bound weakly in binding pocket, such as the longer distance between R103 and the carboxyl group in **(−)**-ABA. Finally, compared with the outward 7′ methyl group in (+)-ABA, the outward bulkier 8′ and 9′ dimethyl group in **(−)**-ABA was adjacent to loop L4 and hindered the closure of it, and thus might weaken the binding to HAB1 (Fig. 3C). Overall, it indicated that PYL3 was a dual ABA receptor, but its binding affinity of **(−)**-ABA was not as strong as that of (+)-ABA.

These results were supported by isothermal titration calorimetry (ITC) experiments. ITC results showed that the binding affinity of **(−)**-ABA to PYL3 was less than that of (+)-ABA [12] or (±)-ABA mixture (Fig. 3E, S5D, S5E), convincingly confirming the weaker binding ability of **(−)**-ABA compared with (+)-ABA. These results were consistent with the previous report that (+)-ABA was eight times more effective than **(−)**-ABA at promoting inhibition of HAB1 mediated by PYL5 [19].

The above analysis showed that unnatural but bioactive enantiomer **(−)**-ABA functioned as an agonist in ABA signaling. To define the residues of PYL3 that underlay **(−)**-ABA binding, important residues involved in binding **(−)**-ABA were mutated. Contrast to wild type PYL3, K79A, F81A, H139A, Y144A, F188A and V192A/V193A mutants could not bind HAB1 and lost the inhibition on HAB1 in the presence of **(−)**-ABA (Fig. 3F). However, most of the above mutants still inhibited HAB1 in the presence of (+)-ABA [12]. Therefore, the binding of **(−)**-ABA to PYL3 was more sensitive to its surrounding residues compared with (+)-ABA. It indicated that the **(−)**-ABA was not in an optimal binding mode, and slightly changing the binding environment may greatly affect its binding. It also meant that the PYLs members might have different binding affinities due to their variable residues.

A previous study [26] showed that the 7′-methyl group was absolutely required for **(−)**-ABA bioactivity, which can be structurally explained since 7′-methyl group was a key residue for ligand binding, forming a hydrophobic interaction with H139 and a hydrophobic interaction network with V134 and L141 (Fig. 3D, S5B). Accordingly, H139A mutant would be expected to disrupt these interactions and thus impair the binding of **(−)**-ABA. Consistent with this observation, we found that H139A mutant lost binding ability and substantially decreased inhibiting ability (Fig. 3F).

### Steric Hindrance Affects the Stereospecificity of PYLs to ABA Enantiomers

**Figure 4:**
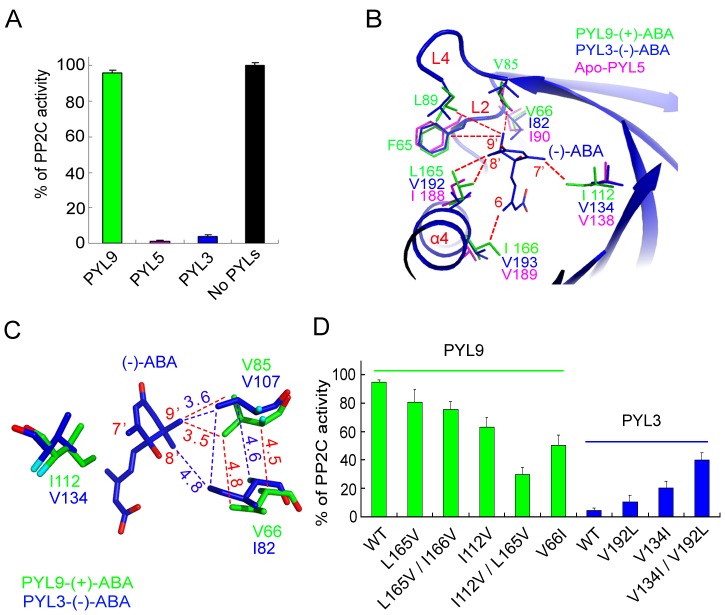
The stereospecificity of PYLs to (+)-ABA or (−)-ABA. (**A**) The different inhibitory efficiency of **(−)**-ABA by PYL3, PYL5 or PYL9. The concentrations of PYLs, HAB1 and **(−)**-ABA were 1 µM, 0.5 µM and 10 µM, respectively. (**B**) Superposition of apo-PYL5, PYL3-**(−)**-ABA and PYL9-(+)-ABA indicated that the major variant residues underlain the favour of PYL binding **(−)**-ABA. Two bulk side chains of I112 and L165 in PYL9 seriously collided to 7′ and 8′ methyl groups of **(−)**-ABA, respectively. The stereo constraints were vanished in PYL5 because of two corresponding small side chains (also seen Fig.S1). (**C**). The mutation V66I in PYL9 would give a strong coordination with 8′ and 9′ methyl groups in **(−)**-ABA through a strong hydrophobic network with surrounding residue V85. (**D**) PYL9 and PYL3 mutants were engineered to gain and cripple the binding of **(−)**-ABA, respectively.

The aforementioned results (Fig. 1C) and other experiments [13] showed that PYL9 had a strong preference for (+)-ABA while PYL5 and PYL3 could bind both ABA enantiomers. We firstly checked the **(−)**-ABA mediated inhibitions of HAB1 by PYL3, PYL5 and PYL9 and confirmed them (Fig. 4A). Then structures of apo-PYL5, PYL3-**(−)**-ABA and PYL9-(+)-ABA were superimposed to insight the stereospecificity of PYLs to both ABA enantiomers., The 7′-methyl of (+)-ABA was confined in a very tight space in PYLs, which was coordinated by bulk residues such as F65, L89 and L165 of PYL9 (Fig. S2B, S1). On the other hand, the pocket ingeniously arranged a bigger room to accommodate the 8′, 9′ dimethyl of (+)-ABA, for example, the bulky residue I112 of PYL9 was involved in direct binding 8′ methyl group of (+)-ABA, respectively (Fig. S2B, S1). Two salt bridges and several hydrogen bonds further constrained the flexibility of (+)-ABA (Fig. S2B).

The **(−)**-ABA imitated the ring and the tail orientations of (+)-ABA (Fig. 3D). However, due to the confinement of 9′ methyl group by the coordination of several bulk residues on the entrance of the pocket of PYLs, the 7′ and 8′ methyl groups of **(−)**-ABA were uncomfortable with the bulk side chains, such as I112 and L165 of PYL9, which make **(−)**-ABA incompatible in the pocket for the strong steric constraints (Fig. 4B). On the contrary, the corresponding two residues in PYL5 and PYL3 were smaller, and thus allowed **(−)**-ABA to dock into the binding cavity (Fig. 4B).

Therefore, the major variable residues surrounding the mono-methyl and di-methyl of ABA cyclohexene ring maybe underlay the preference of PYL binding ABA enantiomers (Fig. 4A, 4B). To testify our analysis, several mutations were introduced into PYL9 according to the corresponding residues in PYL3. As expected, L165V mutation slightly potentiated the binding of **(−)**-ABA (Fig. 4D). In addition, the inhibitory activity by L165V/I166V double mutant was almost the same as L165V mutant, implicating that I166 did not play an important role in **(−)**-ABA recognition. However, the phosphatase activity of HAB1 was obviously inhibited by 40% in the I112V mutant. Furthermore, the PYL9 I112V/L165V double mutant gained significant inhibitory ability (Fig. 4D).

We also performed loss-of-function by introducing larger residues to PYL3, the V134I, V192L and V134I/V192L mutants were engineered to weaken the binding of **(−)**-ABA. Single mutation V192L or V134I attenuated the inhibitory ability (Fig. 4D). The V134I/V192L double mutant crippled the interaction with **(−)**-ABA. These results consistently demonstrated that the exclusive preference of PYLs to (+)-ABA could be abrogated when the two residues involved in directly binding 7′ and 8′ methyl groups of (+)-ABA were smaller, such as those in PYL4, PYL5 and PYL6.

### Hydrophobic Interaction also Affects the Stereospecificity of PYLs to ABA Enantiomers

Surprisingly, we also found the two bulk side chains, corresponding to I112 and L165 of PYL9, existed in PYL8 and PYL10 (Fig. S1). On the contrary to PYL9, PYL8 and PYL10 could inhibited PP2C in the presence of **(−)**-ABA. Although their ability are weaker compared to PYL3 and PYL5 (Fig. 1C). Therefore, there might have another factor to influence PYLs binding **(−)**-ABA. To know the reason, we carefully checked the sequence alignment of all PYLs and found that V66 in PYL9 was not conserved and smallest (Fig. S1). Due to the steric constraint by F65 and L165 and L89, the V66 was 6.8 Å and 6.9 Å far away from 8′,9′ methyl groups of **(−)**-ABA, respectively (Fig. 4B and 4C). Therefore V66 is less possible to contribute the binding of **(−)**-ABA. However, when V66 is mutated to a larger isoleucine, same as that in PYL8 or PYL10, the distance between V66I and 8′,9′ methyl groups of **(−)**-ABA would become about 4.8 and 5.0 Å, respectively (Fig. 4C). Because the distance is in the edge of hydrophobic interaction and thus the direct hydrophobic interaction between them would be weaker. However, V85 is close to V66 and 9′ methyl group of **(−)**-ABA. The distance between V85 and V66 is decrease from 4.8 Å to 3.4 Å when V66 is mutated to isoleucine. Therefore V66I mutation would give a strong coordination with 9′ methyl group in **(−)**-ABA through a strong hydrophobic network with surrounding residue V85 (Fig. 4B, 4C). As expected, the phosphatase activity of HAB1 was obviously inhibited by 50% in the PYL9 V66I mutant in the presence of **(−)**-ABA, while no inhibitory is observed in the wild type PYL9 (Fig. 4D). Therefore, hydrophobic interaction through indirect interaction with 8′, 9′ methyl groups of **(−)**-ABA also contribute the stereospecificity of PYLs to ABA enantiomers.

## Discussion

Our study systematically characterized PYR/PYL/RCAR (PYLs) in the presence of **(−)**-ABA (Fig. 1C). Generally, monomeric PYLs have higher inhibitory ability to inhibit HAB1 than dimeric PYLs [11], which is consistent with in the presence of (+)-ABA. All the tested PYLs can bind (+)-ABA, whereas different PYLs have various binding ability to inhibit PP2C in the presence of **(−)**-ABA. Therefore, PYLs have different stereospecificity of ABA. After extensive trials, we successfully determined structures of PYL3-**(−)**-ABA at high resolution. To the best of our knowledge, we solved the complex structure containing **(−)**-ABA for the first time. Our biochemical and structural data confirmed that several PYLs, at least PYL3, have dual selectivity. In addition, structural analysis elucidated the principle for stereospecificity of PYLs to ABA enantiomer. Steric hindrance and hydrophobic interaction are the two key factors in determining the stereospecificity of ABA. Structural-based gain-of-function and loss-of-function mutations confirmed that the two key factors involved in direct or indirect binding 7′ and 8′, 9′ methyl groups of ABA were responsible for the preference of PYLs to both enantiomers. After comparing the inhibitory abilities by PYLs in the presence of **(−)**-ABA (Fig. 1C), it was deduced that steric hindrance is major factor in this two factors. There are two reasons: One is the inhibitory ability of PYL8 or PYL10 is weaker than PYL3 or PYL5 (Fig. 1C). The other is the PYL9 I112V/L165V double mutant gained higher inhibitory ability than the PYL9 V66I mutant (Fig. 4D).

So far, no structure from subfamily I has been reported [4], our studies presented the first PYL9-(+)-ABA complex structure (Fig. 2A), which shared a similar arrangement with other solved PYLs. However, one disulphide bond was found between C34 and C159 on the molecular surface (Fig. 2B). Sequence alignment of all PYLs indicated that the disulphide bond in PYL9 might be shared by a subclass comprised of PYL4, PYL6 to PYL10 (Fig. S1), in agreement with the corresponding disulphide bond in PYL10 structures (Fig. S3B,C). In addition, the non-conserved C29 was also possible to form a disulphide bond with C159 or C34 due to the flexibility of loop L1 (Fig. 2D), which caused random aggregation (Fig. S2E). Similarly, higher temperature factor of α1 also existed in the PYL3-**(−)**-ABA structures (Fig. S5A). This flexibility was also observed in the structures of PYL10 [27], [28]. The loop L1 and α1 in apo-PYL10 maintained the conformation of those in PYL9, but were apparently different from those in PYL10-HAB1 (Fig. S3B,C). The disulphide bond existed in apo-PYL10, PYL10-(+)-ABA and PYL9-(+)-ABA, but it disappeared in PYL10-HAB1 structure. It raised a question whether it was necessary to interrupt the disulphide bond for PYLs to inhibit PP2Cs? *In vitro*, the C29S, C34S or C159S mutations did not significantly affect the inhibition of HAB1 by PYL9 in the presence of (+)-ABA, which implied that the disulphide bond was little linked with the inhibition of PP2Cs activity (Fig. S3A). However, we can not simply deny that the disulphide bond or cysteines may execute some function *in vivo*. Therefore, whether the disulphide bond participates in ABA signaling remains to be elucidated by further genetic and biochemical study.

Since the discovery of ABA [3], [13] in 1960s, the biological and biochemical activities of unnatural **(−)**-ABA have been extensively investigated, such as seed germination [14], plant tissues growth [13] and stomatal closure. Two mechanisms, Milborrow’s long-standing hypothesis [3], [13] and dual selectivity of ABA receptors [14], were proposed to explain the bioactivity of **(−)**-ABA. Our results deepened the cognition of **(−)**-ABA enantiomer. First of all, our results did not support Milborrow’s long-standing hypothesis [3], [13]. In fact, **(−)**-ABA was clearly visualized in our PYL3-**(−)**-ABA structure and its position was obviously different from that of (+)-ABA (Fig. 3C and 3D). Besides, after flipping **(−)**-ABA, the positions of 7′ and 8′,9′ methyl groups differed greatly from those of (+)-ABA because the cyclohexene was not a true plane (Fig. S5C). Thus the binding environment and residues in the pocket of PYLs would be affected greatly by the methyl groups. In addition, the dramatic contrast of binding **(−)**-ABA between PYL5 and PYL9 also denied the “flip” hypothesis, which supported the hypothesis of dual selectivity of ABA receptor [29]. Our result provided important insights into the different biological activity of unnatural **(−)**-ABA *in vivo*, such as stomatal closure [30] and seed germination [14]. However, there are still some questions to be elucidated, for example, applied **(−)**-ABA can trigger biosynthesis of natural (+)-ABA [16], stronger morphogenic effects and lower optimal concentration compared with (+)-ABA [14].

The *Arabidopsis* genome encodes 13 PYLs and 9 clade-A PP2Cs. Phosphatase activity of different combination of PP2Cs and PYLs regulated by ligands differed greatly [31]. Therefore appropriate ligands would modulate and fine-tune ABA responses. To date, there is no other selective ABA analogs except **(−)**-ABA and pyrabactin. Our data suggest that chemical modification of **(−)**-ABA at three methyl groups will enable the design of selective agonists to modulate and fine-tune ABA receptors. The recognition mechanism also shed light on the potential engineering of transgenic plants that may exhibit enhanced tolerance to environmental stress. In summary, our study systematically characterized PYR/PYL/RCAR (PYLs) in the presence of **(−)**-ABA and presented the first complex structure of PYLs with **(−)**-ABA. Biochemical and structural data confirmed that some PYLs, at least PYL3, had the dual selectivity. In addition, the two key factors involved in direct and indirect binding 7′ and 8′, 9′ methyl groups of ABA were responsible for the stereospecificity of PYLs to ABA. Our study provides novel insights into the design of selective ABA agonists and paves the way for generation of the transgenic crop with high yield and tolerance of abiotic stresses.

### Accession Numbers

Coordinates and structure factors are deposited in the Protein Data Bank under accession numbers 3OQU, 4JDL and 4JDA.

## Supporting Information

Figure S1 **Primary sequence alignment of PYL9 (residues 26–169) with other PYLs members.** The sequence alignment was generated by ClustalW [1]. This figure was made by the program ALSCRIPT [2]. All columns with similarities in physico-chemical properties were shown in yellow background for black character of residues. The identical residues in red background were represented in white characters. The secondary structures of PYL9 were shown above the sequence of PYL9, composed of four helices and seven β strands. The nomenclature according to the previous publications was colored blue, for example loop L4 was also known as Gate [3] or CL2 [4] and loop L5 was also known as Latch [3] or CL3 [4]. Three variable residues responsible for the stereospecificity of PYLs to ABA enantiomers (seen Figure 4B, 2C) were labeled with rectangles. The four residues in PYLs involved in coordinating the ABA (see Figure 4B) were marked with green triangles under PYL13.
(DOC)

Figure S2 **Crystal structure and biochemical properties of PYL9. (A)** PYL9-(+)-ABA crystals and their enlarged picture (right) appeared after about 2 weeks in heavy protein precipitate. **(B)** The ligand (+)-ABA was encaged in a conserved pocket formed by several hydrogen bonds, two salt bridges and abundant hydrophobic interactions. The key residues in binding (+)-ABA were shown in red. **(C)** Both PYL9 and PYL5 were monomer by size exclusive chromatography (SEC). **(D)** Crosslinking results of apo-PYL9 at different EGS concentrations were visualized by SDS-PAGE and then Coomassie Brilliant Blue staining, which showed that PYL9 was monomer in solution. Our previous study showed the apo-PYL3 mainly existed in dimer state as control [5]. **(E)** The monomeric PYL9 was not prone to aggregate. The eluant for 6×His tagged PYL9 from Profinity™ IMAC Ni-Charged Resin column was subjected to SEC and the factions were detected by SDS-PAGE and then Coomassie Brilliant Blue staining. The monomeric fractions picked from the first round of SEC were subjected once more. The curve illustrated that monomeric PYL9 was not prone to oligomerize.
(DOC)

Figure S3 **The interchangeable disulphide bond and cysteines of PYL9. (A)** The disulphide bond had no obvious impact on the inhibition of HAB1 in the presence of (+)-ABA. Each reaction was repeated at least three times and the error bars indicated standard deviations. **(B)** The loop L4 in apo-PYL10 was in a closed state like that in PYL10-(+)-ABA. Superposition of apo-PYL10 (PDB: 3UQH, green) and PYL10-(+)-ABA (PDB: 3R6P, cyan). The (+)-ABA, disulphide bonds and loop L4 were shown in yellow, red and blue, respectively. **(C)** Superposition of apo-PYL10 (PDB: 3RT2, green) and PYL10-HAB1 (PDB: 3RT0, PYL10, orange; HAB1, magenta), the disulphide bond in apo-PYL10 circled in black was enlarged in the right panel and the L4 in both structures were circled in blue. Interestingly, disulphide bond had 50% occupancy in apo-PYL10 and it was not observed in PYL10-HAB1 structure (right panel). The conformations of two α1 helixes were obviously different. Together with the information in Fig. 3D, it implied that the disulphide bond in PYL9 was dynamic.
(DOC)

Figure S4 **The structure of apo-PYL5.** (**A**) X-ray diffraction image from apo-PYL5 crystal. The crystal diffraction data were determined to be highly merohedral-twinned with the twinning operator (h, -h-k, -l) and a twinning fraction of 0.478, as judged by cumulative intensity distribution calculated with program Phenix [6]. (**B**) There were three protomers in an asymmetric unit of apo-PYL5. **(C)** Superposition of three protomers of apo-PYL5. Both loops L4 of chain A and chain B were disappeared while loop L4 of chain C had clear electron density. On the contrary, electron density for both loops L2 of chain A and chain B was clear while it was not visual for L2 of chain C (seen Fig. 3A).
(DOC)

Figure S5 **Structural characterization of PYL3-(−)ABA**. (**A**) All residues in PYL3 for **(−)**-ABA binding are of low B-factors. Overall B-factors of the PYL3-**(−)**-ABA structures, color-coded on the basis of the calculated B-factors, the colors range from blue to red corresponding to increasing fluctuations. (**B**) The ligands **(−)**-ABA and (+)-ABA (PDB:4DSC) were encaged in PYL3 conserved pocket formed by several kinds of interactions. (**C**) Aligned structures of **(−)**-ABA and (+)-ABA based on the cyclohexene plane. (**D**) (±)-ABA was injected to PYL3. (**E**) Buffer A (20 mM Tris-HCl pH 8.0 and 150 mM NaCl) was taken as the control.
(DOC)

File S1 **Supplementary Experimental Procedures.**
(DOC)
