# Supplementary material for: Structural Insights into the Abscisic Acid Stereospecificity by the ABA Receptors PYR/PYL/RCAR
Source: PLoS One. 2013 Jul 2;8(7):e67477. doi: 10.1371/journal.pone.0067477 (PMC3699650; doi:10.1371/journal.pone.0067477)

**Figure S1. Primary sequence alignment of PYL9 (residues 26-169) with other PYLs members.** The sequence alignment was generated by ClustalW . This figure was made by the program ALSCRIPT . All columns with similarities in physico-chemical properties were shown in yellow background for black character of residues. The identical residues in red background were represented in white characters. The secondary structures of PYL9 were shown above the sequence of PYL9, composed of four helices and seven β strands. The nomenclature according to the previous publications was colored blue, for example loop L4 was also known as Gate or CL2 and loop L5 was also known as Latch or CL3 . Three variable residues responsible for the stereospecificity of PYLs to ABA enantiomers (seen Figure 4B, 2C) were labeled with rectangles. The four residues in PYLs involved in coordinating the ABA (see Figure 4B) were marked with green triangles under PYL13.


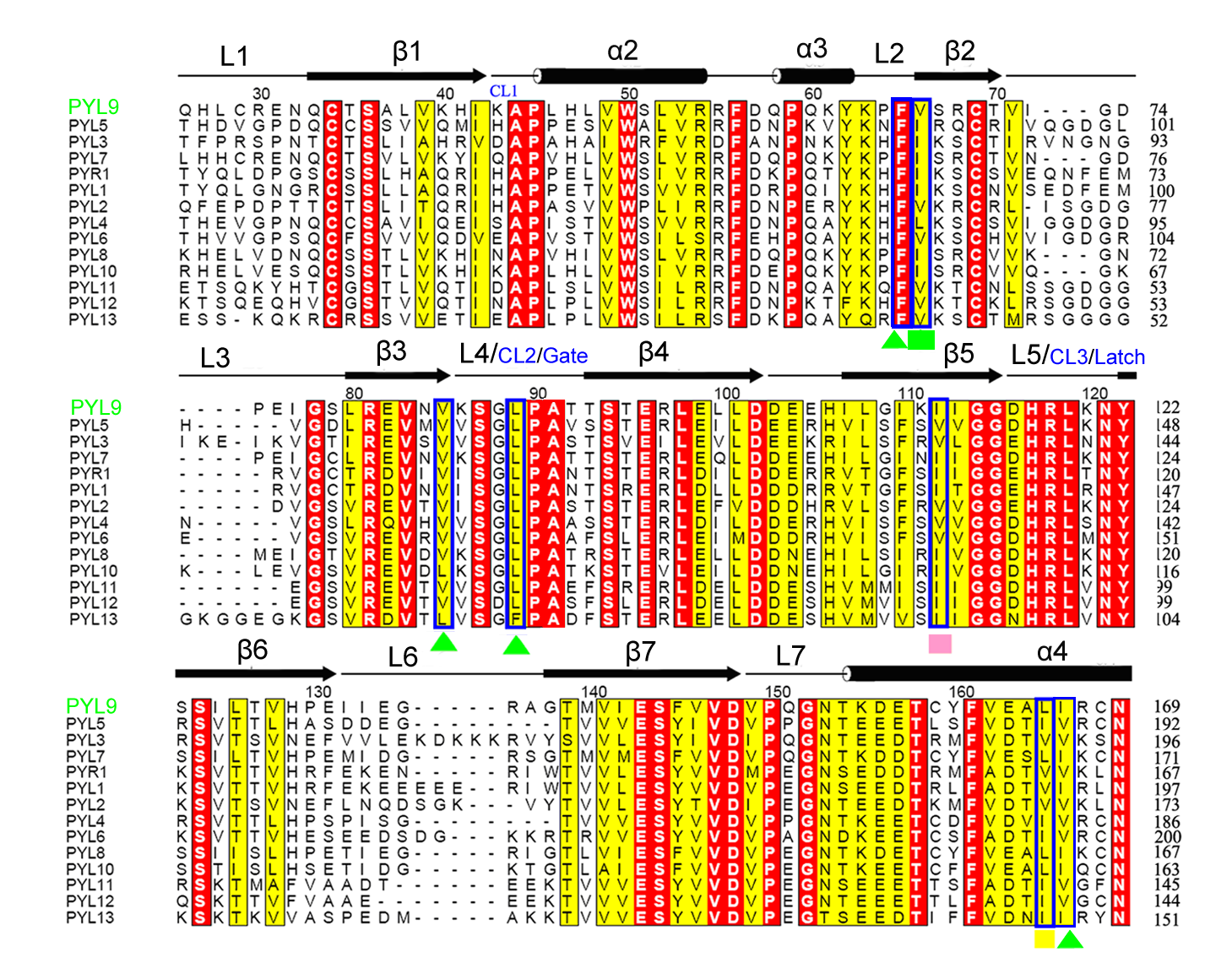

Supplement: Figure S1 — Primary sequence alignment of PYL9 (residues 26–169) with other PYLs members. The sequence alignment was generated by ClustalW [1]. This figure was made by the program ALSCRIPT [2]. All columns with similarities in physico-chemical properties were shown in yellow background for black character of residues. The identical residues in red background were represented in white characters. The secondary structures of PYL9 were shown above the sequence of PYL9, composed of four helices and seven β strands. The nomenclature according to the previous publications was colored blue, for example loop L4 was also known as Gate [3] or CL2 [4] and loop L5 was also known as Latch [3] or CL3 [4]. Three variable residues responsible for the stereospecificity of PYLs to ABA enantiomers (seen Figure 4B, 2C) were labeled with rectangles. The four residues in PYLs involved in coordinating the ABA (see Figure 4B) were marked with green triangles under PYL13. (DOC) [file pone.0067477.s001.doc]
