# Supplementary material for: Structural Insights into the Abscisic Acid Stereospecificity by the ABA Receptors PYR/PYL/RCAR
Source: PLoS One. 2013 Jul 2;8(7):e67477. doi: 10.1371/journal.pone.0067477 (PMC3699650; doi:10.1371/journal.pone.0067477)

**Figure S2. Crystal structure and biochemical properties of PYL9. (A)** PYL9-(+)-ABA crystals and their enlarged picture (right) appeared after about 2 weeks in heavy protein precipitate. **(B)** The ligand (+)-ABA was encaged in a conserved pocket formed by several hydrogen bonds, two salt bridges and abundant hydrophobic interactions. The key residues in binding (+)-ABA were shown in red. **(C)** Both PYL9 and PYL5 were monomer by size exclusive chromatography (SEC). **(D)** Crosslinking results of apo-PYL9 at different EGS concentrations were visualized by SDS-PAGE and then Coomassie Brilliant Blue staining, which showed that PYL9 was monomer in solution. Our previous study showed the apo-PYL3 mainly existed in dimer state as control [5]. **(E)** The monomeric PYL9 was not prone to aggregate. The eluant for 6×His tagged PYL9 from Profinity^TM^ IMAC Ni-Charged Resin column was subjected to SEC and the factions were detected by SDS-PAGE and then Coomassie Brilliant Blue staining. The monomeric fractions picked from the first round of SEC were subjected once more. The curve illustrated that monomeric PYL9 was not prone to oligomerize.


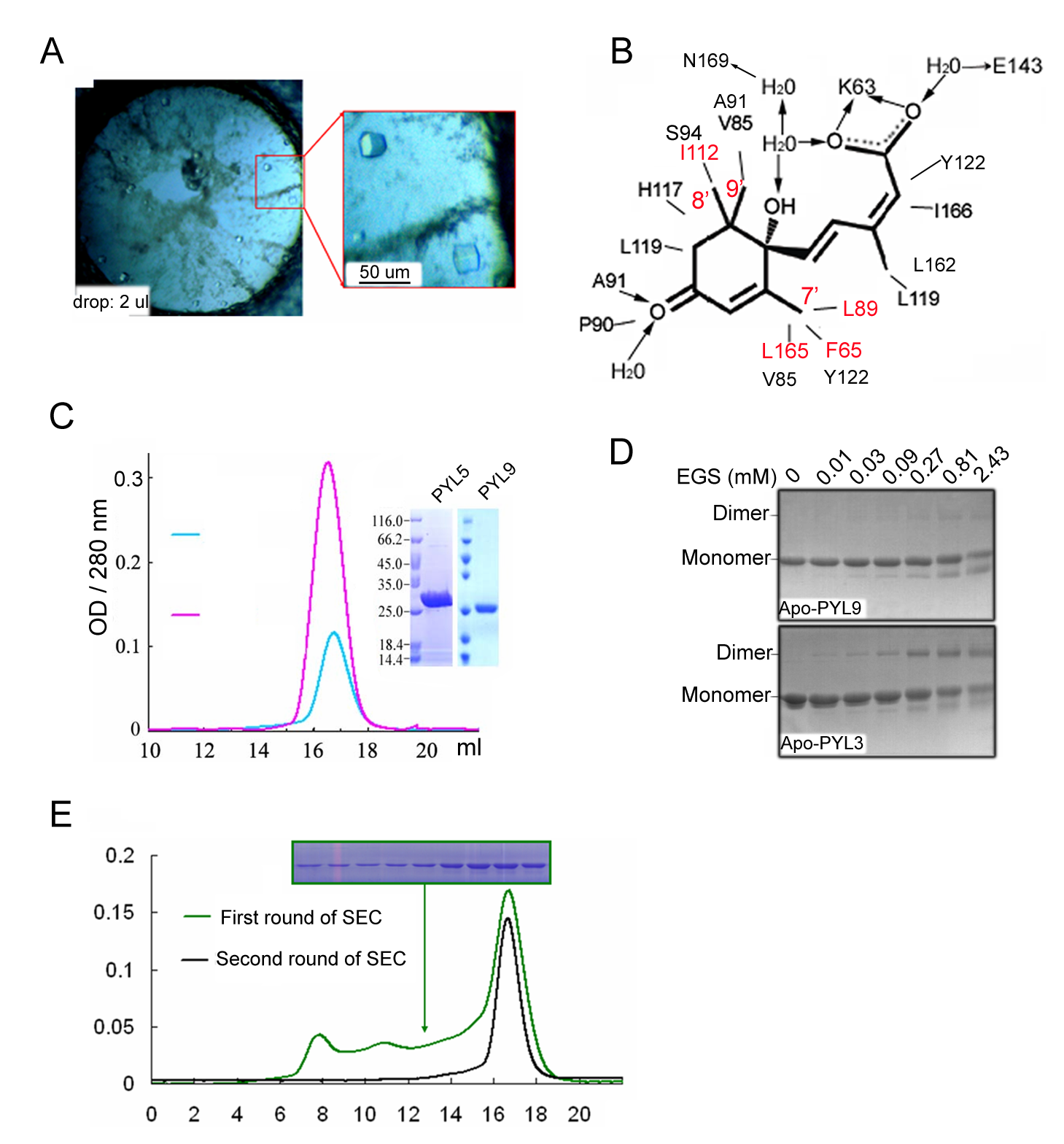

Supplement: Figure S2 — Crystal structure and biochemical properties of PYL9. (A) PYL9-(+)-ABA crystals and their enlarged picture (right) appeared after about 2 weeks in heavy protein precipitate. (B) The ligand (+)-ABA was encaged in a conserved pocket formed by several hydrogen bonds, two salt bridges and abundant hydrophobic interactions. The key residues in binding (+)-ABA were shown in red. (C) Both PYL9 and PYL5 were monomer by size exclusive chromatography (SEC). (D) Crosslinking results of apo-PYL9 at different EGS concentrations were visualized by SDS-PAGE and then Coomassie Brilliant Blue staining, which showed that PYL9 was monomer in solution. Our previous study showed the apo-PYL3 mainly existed in dimer state as control [5]. (E) The monomeric PYL9 was not prone to aggregate. The eluant for 6×His tagged PYL9 from Profinity™ IMAC Ni-Charged Resin column was subjected to SEC and the factions were detected by SDS-PAGE and then Coomassie Brilliant Blue staining. The monomeric fractions picked from the first round of SEC were subjected once more. The curve illustrated that monomeric PYL9 was not prone to oligomerize. (DOC) [file pone.0067477.s002.doc]
