# Supplementary material for: Structural Insights into the Abscisic Acid Stereospecificity by the ABA Receptors PYR/PYL/RCAR
Source: PLoS One. 2013 Jul 2;8(7):e67477. doi: 10.1371/journal.pone.0067477 (PMC3699650; doi:10.1371/journal.pone.0067477)

**Figure S3. The interchangeable disulphide bond and cysteines of PYL9. (A)** The disulphide bond had no obvious impact on the inhibition of HAB1 in the presence of (+)-ABA. Each reaction was repeated at least three times and the error bars indicated standard deviations. **(B)** The loop L4 in apo-PYL10 was in a closed state like that in PYL10-(+)-ABA. Superposition of apo-PYL10 (PDB: 3UQH, green) and PYL10-(+)-ABA (PDB: 3R6P, cyan). The (+)-ABA, disulphide bonds and loop L4 were shown in yellow, red and blue, respectively. **(C)** Superposition of apo-PYL10 (PDB: 3RT2, green) and PYL10-HAB1 (PDB: 3RT0, PYL10, orange; HAB1, magenta), the disulphide bond in apo-PYL10 circled in black was enlarged in the right panel and the L4 in both structures were circled in blue. Interestingly, disulphide bond had 50% occupancy in apo-PYL10 and it was not observed in PYL10-HAB1 structure (right panel). The conformations of two α1 helixes were obviously different. Together with the information in Fig.3D, it implied that the disulphide bond in PYL9 was dynamic.


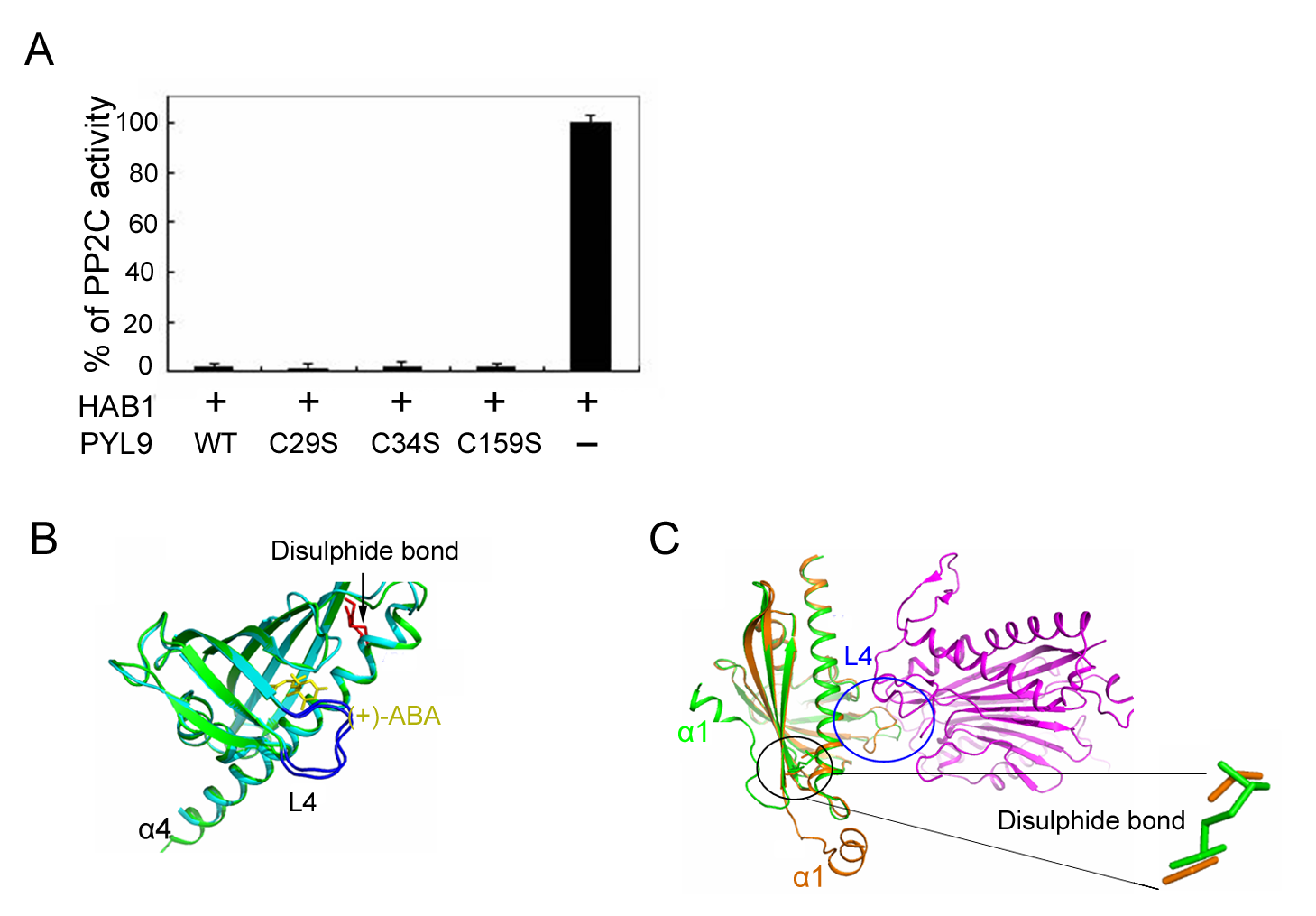

Supplement: Figure S3 — The interchangeable disulphide bond and cysteines of PYL9. (A) The disulphide bond had no obvious impact on the inhibition of HAB1 in the presence of (+)-ABA. Each reaction was repeated at least three times and the error bars indicated standard deviations. (B) The loop L4 in apo-PYL10 was in a closed state like that in PYL10-(+)-ABA. Superposition of apo-PYL10 (PDB: 3UQH, green) and PYL10-(+)-ABA (PDB: 3R6P, cyan). The (+)-ABA, disulphide bonds and loop L4 were shown in yellow, red and blue, respectively. (C) Superposition of apo-PYL10 (PDB: 3RT2, green) and PYL10-HAB1 (PDB: 3RT0, PYL10, orange; HAB1, magenta), the disulphide bond in apo-PYL10 circled in black was enlarged in the right panel and the L4 in both structures were circled in blue. Interestingly, disulphide bond had 50% occupancy in apo-PYL10 and it was not observed in PYL10-HAB1 structure (right panel). The conformations of two α1 helixes were obviously different. Together with the information in Fig. 3D, it implied that the disulphide bond in PYL9 was dynamic. (DOC) [file pone.0067477.s003.doc]
