# Supplementary material for: Structural Insights into the Abscisic Acid Stereospecificity by the ABA Receptors PYR/PYL/RCAR
Source: PLoS One. 2013 Jul 2;8(7):e67477. doi: 10.1371/journal.pone.0067477 (PMC3699650; doi:10.1371/journal.pone.0067477)
Supplement: Figure S4 — The structure of apo-PYL5. (A) X-ray diffraction image from apo-PYL5 crystal. The crystal diffraction data were determined to be highly merohedral-twinned with the twinning operator (h, -h-k, -l) and a twinning fraction of 0.478, as judged by cumulative intensity distribution calculated with program Phenix [6]. (B) There were three protomers in an asymmetric unit of apo-PYL5. (C) Superposition of three protomers of apo-PYL5. Both loops L4 of chain A and chain B were disappeared while loop L4 of chain C had clear electron density. On the contrary, electron density for both loops L2 of chain A and chain B was clear while it was not visual for L2 of chain C (seen Fig. 3A). (DOC) [file pone.0067477.s004.doc]

**Figure S4. The structure of apo-PYL5.** (**A**) X-ray diffraction image from apo-PYL5 crystal. The crystal diffraction data were determined to be highly merohedral-twinned with the twinning operator (h, -h-k, -l) and a twinning fraction of 0.478, as judged by cumulative intensity distribution calculated with program Phenix [6]. (**B**) There were three protomers in an asymmetric unit of apo-PYL5. **(C)** Superposition of three protomers of apo-PYL5. Both loops L4 of chain A and chain B were disappeared while loop L4 of chain C had clear electron density. On the contrary, electron density for both loops L2 of chain A and chain B was clear while it was not visual for L2 of chain C (seen Fig.3A).

**
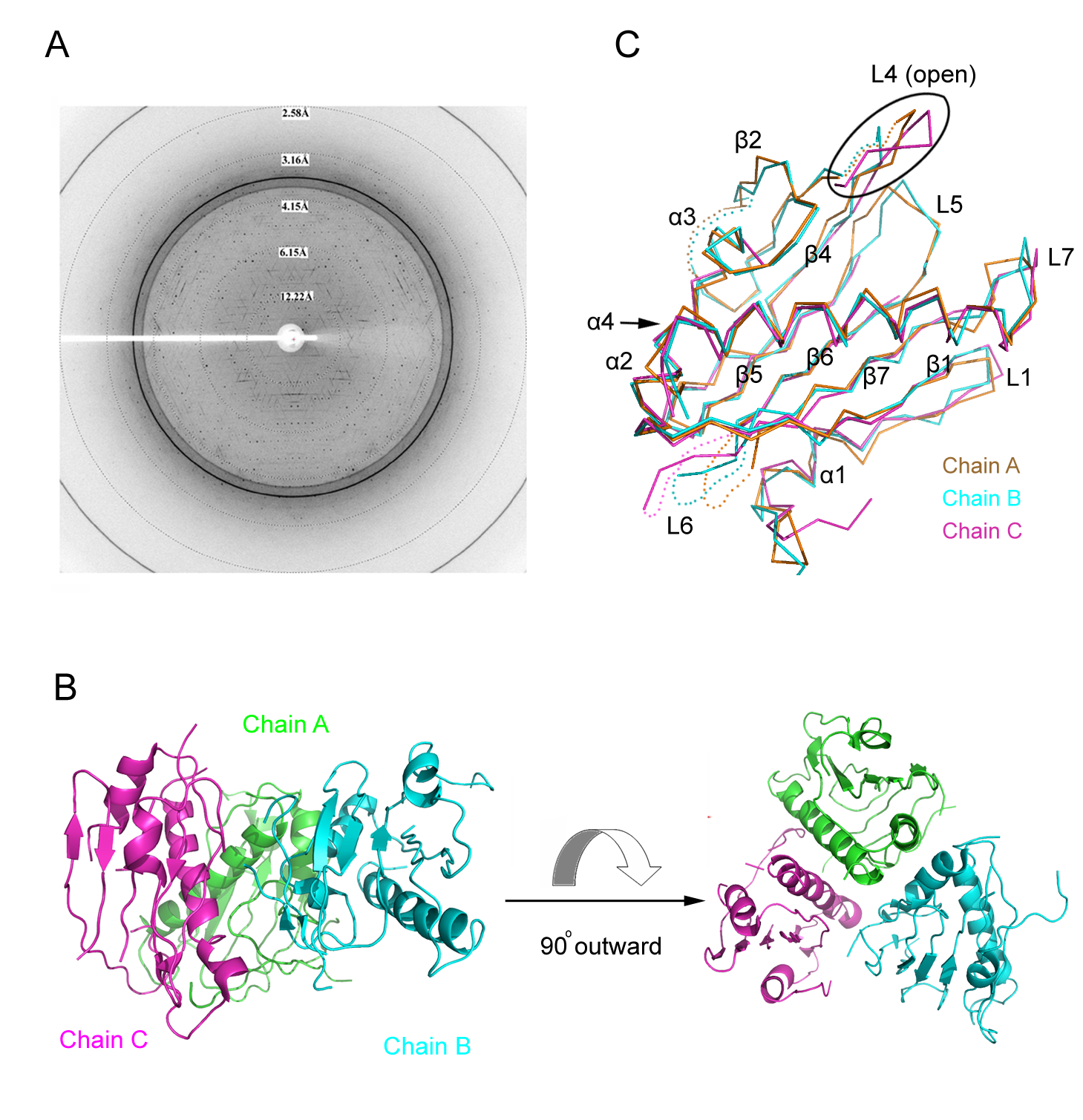
**
