# Supplementary material for: Structural Insights into the Abscisic Acid Stereospecificity by the ABA Receptors PYR/PYL/RCAR
Source: PLoS One. 2013 Jul 2;8(7):e67477. doi: 10.1371/journal.pone.0067477 (PMC3699650; doi:10.1371/journal.pone.0067477)
Supplement: Figure S5 — Structural characterization of PYL3-(−)ABA. (A) All residues in PYL3 for (−)-ABA binding are of low B-factors. Overall B-factors of the PYL3-(−)-ABA structures, color-coded on the basis of the calculated B-factors, the colors range from blue to red corresponding to increasing fluctuations. (B) The ligands (−)-ABA and (+)-ABA (PDB:4DSC) were encaged in PYL3 conserved pocket formed by several kinds of interactions. (C) Aligned structures of (−)-ABA and (+)-ABA based on the cyclohexene plane. (D) (±)-ABA was injected to PYL3. (E) Buffer A (20 mM Tris-HCl pH 8.0 and 150 mM NaCl) was taken as the control. (DOC) [file pone.0067477.s005.doc]

**Figure S5.** **Structural characterization of PYL3-(-)ABA**. (**A**) All residues in PYL3 for (-)-ABA binding are of low B-factors. Overall B-factors of the PYL3-(-)-ABA structures, color-coded on the basis of the calculated B-factors, the colors range from blue to red corresponding to increasing fluctuations. (**B**) The ligands (-)-ABA and (+)-ABA (PDB：4DSC) were encaged in PYL3 conserved pocket formed by several kinds of interactions. (**C**) Aligned structures of (-)-ABA and (+)-ABA based on the cyclohexene plane. (**D**) (±)-ABA was injected to PYL3. (**E**) Buffer A (20 mM Tris-HCl pH 8.0 and 150 mM NaCl) was taken as the control.

**
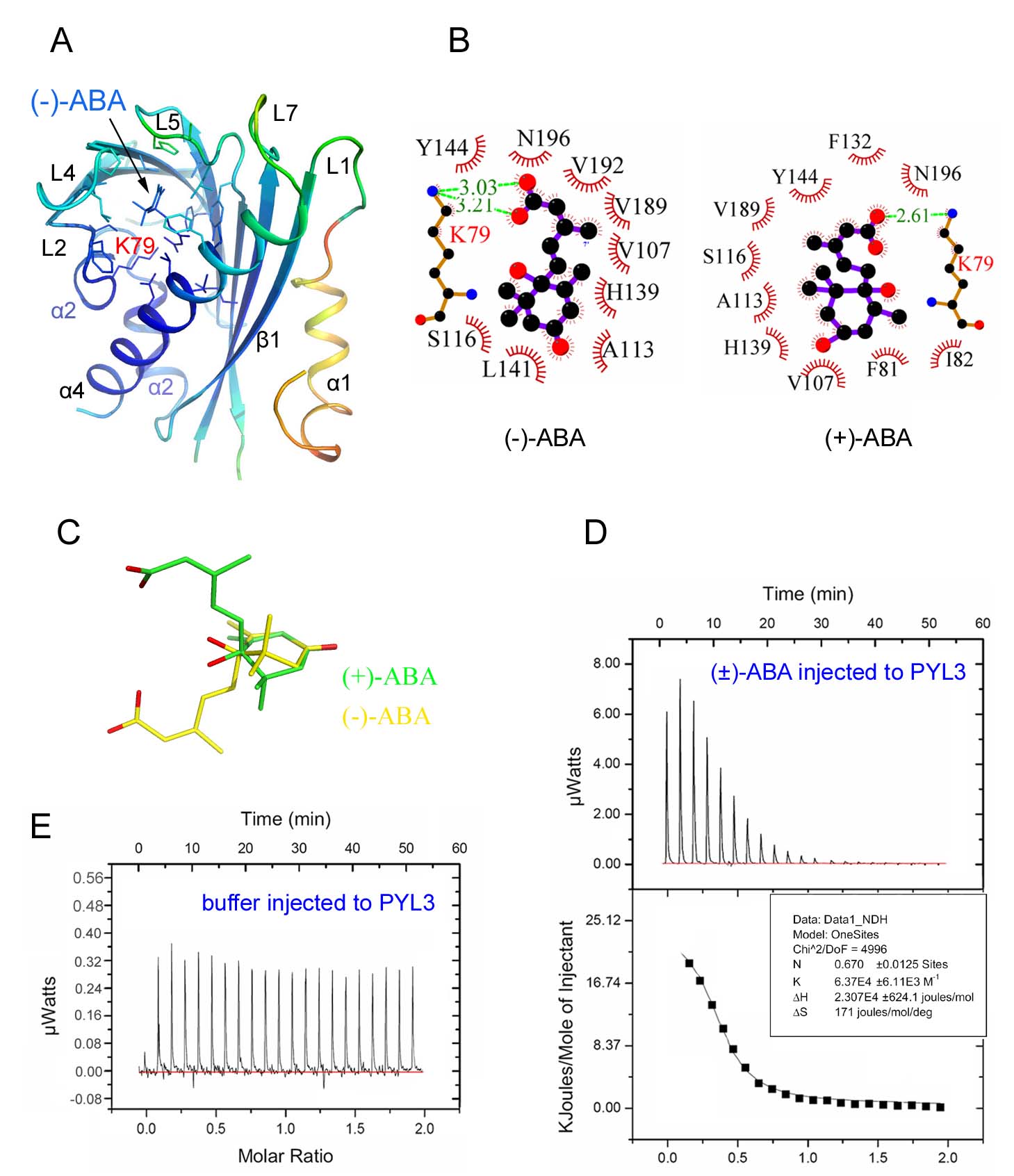
**
