# Supplementary material for: Structural Insights into the Abscisic Acid Stereospecificity by the ABA Receptors PYR/PYL/RCAR
Source: PLoS One. 2013 Jul 2;8(7):e67477. doi: 10.1371/journal.pone.0067477 (PMC3699650; doi:10.1371/journal.pone.0067477)
Supplement: File S1 — Supplementary Experimental Procedures. (DOC) [file pone.0067477.s006.doc]

**Supplementary Experimental Procedures**

**Analytical ultracentrifugation**

Sedimentation velocity (SV) experiments were performed in a Beckman/Coulter XL-I analytical ultracentrifuge using double-sector or six-channel centerpieces and sapphirine windows. An additional protein purification step applied to size exclusion chromatography in buffer containing 20 mM Tris-HCl pH 8.0, 150 mM NaCl was performed before experiments. SV experiments were conducted at 42000 rpm and 10℃ using interference detection and double-sector cells loaded at approximate 0.1 mM for 6×His-PYL9. The buffer composition (density and viscosity) and protein partial specific volume (V-bar) were obtained using the program SEDNTERP (http://www.rasmb.bbri.org/). The SV data were analyzed using the SEDFIT and SEDPHAT programs .

**Cross-linking gel assay**

Ethylene glycol-bis (succinic acid N-hydroxysuccinimide ester) (EGS, Sigma-Aldrich, E-3257) was dissolved in dimethyl sulfoxide to 20 mM. After subjected to size exclusion chromatography with phosphate buffered saline (pH 7.5, 150 mM NaCl), 20 μg purified apo-PYL9 protein or apo-PYL3 protein (a known dimeric protein as control) was added to 100 μl of reaction volume with different EGS concentrations. The mixture was incubated at room temperature for 10 min, and then the reaction was quenched for 10 min by adding 5 µl of 1 M Tris-Glycine (pH 7.5). A quarter volume of 5×SDS-polyacrylamide gel electrophoresis sample buffer was added to the reaction mixture, and a small amount was analyzed on a 10% SDS polyacrylamide gel and visualized by Coomassie Brilliant Blue staining.

**Size exclusion chromatography (SEC)**

6×His-PYL9 protein, 6×His-PYL3, 6×His-PYL5 and their mutants were applied to Superdex 200 HR10/300 GL (GE Healthcare) in a buffer containing 20 mM Hepes pH 7.5 and 150 mM NaCl. The protein after size exclusion chromatography was use for the experiment of Phosphatase activity assay. The fractions were visualized by SDS-PAGE followed by Coomassie Brilliant Blue staining to estimate their purity.

**GST-mediated pulldown assay**

200 μg GST-fusion HAB1 (residue 169-511) protein was immobilized on Glutathione Sepharose 4 FF Resin (GE Healthcare). 50 μg WT or mutant PYL3 proteins, incubated with (-)-ABA, were incubated with the HAB1-bound GST resin for an hour at 25 °C. The resin was then extensively rinsed with buffer (20 mM Tris-HCl pH8.0 and 150 mM NaCl) to wash unbound and non-specific bound proteins. The resin was resuspended with 200 μl of the above buffer containing 10mM GSH in 10 minutes, and was centrifuged at 13,800 rpm (17032g) for 10 minutes. 20 μl of the supernatant was taken out and applied to SDS-PAGE, then visualized with Coomassie Brilliant Blue staining.

**Supplementary References**

1. Larkin MA, Blackshields G, Brown NP, Chenna R, McGettigan PA, et al. (2007) Clustal W and Clustal X version 2.0. Bioinformatics 23: 2947-2948.

2. Barton GJ (1993) ALSCRIPT: a tool to format multiple sequence alignments. Protein Eng 6: 37-40.

3. Melcher K, Ng LM, Zhou XE, Soon FF, Xu Y, et al. (2009) A gate-latch-lock mechanism for hormone signalling by abscisic acid receptors. Nature 462: 602-608.

4. Yin P, Fan H, Hao Q, Yuan X, Wu D, et al. (2009) Structural insights into the mechanism of abscisic acid signaling by PYL proteins. Nat Struct Mol Biol 16: 1230-1236.

5. Adams PD, Afonine PV, Bunkoczi G, Chen VB, Davis IW, et al. (2010) PHENIX: a comprehensive Python-based system for macromolecular structure solution. Acta Crystallogr D Biol Crystallogr 66: 213-221.

6. Zhang X, Zhang Q, Xin Q, Yu L, Wang Z, et al. (2012) Complex Structures of the Abscisic Acid Receptor PYL3/RCAR13 Reveal a Unique Regulatory Mechanism. Structure 20: 780-790.

7. Schuck P (2000) Size-distribution analysis of macromolecules by sedimentation velocity ultracentrifugation and lamm equation modeling. Biophys J 78: 1606-1619.

8. Schuck P (2003) On the analysis of protein self-association by sedimentation velocity analytical ultracentrifugation. Anal Biochem 320: 104-124.
